# The involvement of Th17 inflammation and miR-363-3p in airway epithelial barrier dysfunction

**DOI:** 10.1186/s12931-025-03492-3

**Published:** 2026-01-16

**Authors:** Cecilia Lässer, Elisabeth Ax, Julie Weidner, Sofia Winslow, Hannes Ingelhag, Jenny Calvén, Carina Malmhäll, Zala Jevnikar, Henric Olsson, Madeleine Rådinger

**Affiliations:** 1 Krefting Research Centre, Institute of Medicine at the Sahlgrenska Academy, University of Gothenburg, Gothenburg, Sweden; 2 Translational Science and Experimental Medicine, Research and Early Development, AstraZeneca, Respiratory & Immunology, BioPharmaceuticals R&D, Gothenburg, Sweden

**Keywords:** Airway epithelium, Asthma, Endotypes, MicroRNA, Th17, Inflammation

## Abstract

**Background:**

Impaired airway epithelial barrier function is a pathogenic driver in a subset of individuals with asthma. MicroRNAs, small RNAs that function as post-transcriptional regulators of gene expression, may be involved in the regulation of the airway epithelial barrier. This study aimed to determine if microRNAs cause epithelial barrier dysfunction through the targeting of mRNAs involved in maintaining airway epithelial barrier integrity.

**Methods:**

Primary human bronchial epithelial cells cultured at air–liquid interface were stimulated with cytokines reflecting different asthma endotypes. Barrier integrity was assessed by FITC-labelled dextran flux. Differentially expressed epithelial barrier-related genes and microRNAs were identified by next-generation RNA sequencing and qPCR. Results were validated with microRNA pull-down, treatment with microRNA mimics and antagomirs, respectively, and evaluated in airway samples from subjects with asthma.

**Results:**

A combination of IL-17A and TNFα, mimicking Th17 inflammation, was identified as a strong driver of epithelial barrier disruption, as compared to IL-4 + IL-13, IL-6 + sIL-6R, and TGFβ. Several microRNAs induced by IL-17A and TNFα stimulation were predicted to target barrier-related genes, which exhibited decreased expression in the same model. Of these microRNAs, miR-146a-3p and miR-363-3p were consistently induced in multiple donors. MicroRNA pull-down, overexpression, and knockdown experiments indicated a potential role for miR-363-3p interacting with several barrier-related genes, including *CLDN8*, *PCDH1*, and *PTEN*. Bronchial lavage samples demonstrated an increase of miR-363-3p in individuals with asthma compared to healthy controls, as well as a positive correlation between miR-363-3p and the number of airway eosinophils and neutrophils.

**Conclusions:**

Our results support the role of microRNAs as mediators of cytokine-induced airway epithelial barrier dysfunction. Specifically, miR-363-3p appears to contribute to epithelial damage by targeting and suppressing gene expressions of several key barrier components, including *CLDN8*, *PCDH1*, and *PTEN*, suggesting a novel role for this microRNA in Th17-driven airway disease. A better understanding of microRNA networks and their role in asthma pathogenesis may lead to novel biomarkers and therapeutic targets, which are currently needed for individuals with T2-low asthma and in Th17-driven asthma.

**Supplementary Information:**

The online version contains supplementary material available at 10.1186/s12931-025-03492-3.

## Background

The development of precision medicine approaches for the treatment of asthma requires an understanding of the various molecular mechanisms responsible for disease heterogeneity. Targeting ‘treatable mechanisms or traits’, such as eosinophilia, cough, or ciliary dysfunction, has the potential to drive improvement in a subset of individuals with the disease [1–3]. Disease endotypes encompass a combination of molecular mechanisms and clinical parameters that can be used to define patient subpopulations. The different inflammatory profiles observed in asthma highlight the heterogeneity of the disease. Type 2 (T2) asthma, characterized by Type 2 helper T cells (Th2), innate lymphoid cells type 2 (ILC2s), T2 cytokines, and eosinophils, is the best described and clinically validated endotype [4, 5], whereas T2-low asthma is less studied. T2-low asthma is generally characterized by late onset and an absence of airway eosinophilia but is often associated with neutrophilic inflammation. However, recent studies suggest that in most patients with severe T2-low asthma, the phenotype may change to T2-high during an exacerbation. T2-low asthma contains several subtypes involving other immune responses, such as Type 17 (Th17) asthma, which is associated with Type 17 helper T cell (Th17) cytokines and neutrophils [5–7], Type 1 helper T cell (Th1)-related inflammation, innate immune activation, and interleukin (IL)−6 trans-signaling (IL6TS) [8, 9]. However, these subtypes comprise less defined patient groups. While several biomarkers define T2 asthma, other asthma endotypes are currently identified through the absence or low levels of T2 biomarkers [8]. Furthermore, the standard of care, predominantly consisting of corticosteroids, is aimed mainly at treating individuals with T2 asthma [3, 8], and there is a need for new, improved treatments and biomarkers for individuals with T2-low asthma. One important, but less explored aspect of asthma is airway epithelial barrier dysfunction [10, 11], which is present to varying degrees in inflammatory airway diseases [12, 13]. In asthma, barrier dysfunction may be due to genetics, disease-related factors such as inflammatory cytokines, or environmental exposures such as particulate matter, pollution, and allergens [10, 12, 13]. Changes in cellular composition of the epithelium and the modulation of inter-epithelial junction proteins such as tight junction protein ZO-1 (TJP1), claudins, and β-catenin are hallmarks of barrier dysfunction [11, 13, 14]. Such perturbations may increase susceptibility to injury, obstruct repair processes, and affect the local airway environment through secretion of mediators [12, 13]. These mechanisms play an important role in disease initiation and progression, suggesting that future therapeutics targeting barrier dysfunction may benefit patients with asthma.

One class of molecules likely to be involved in barrier dysfunction are microRNAs (miRNAs), short non-coding RNAs that regulate post-transcriptional gene expression [15]. miRNAs cause decreased protein expression by binding to target mRNAs and either inhibiting their translation or tagging them for degradation [15]. The role of miRNAs in airway diseases, and especially in airway epithelial cell function, is largely unexplored*.* Studies have shown differential expressions of miRNAs in the airway epithelium and in airway-derived samples from individuals with asthma as compared to healthy individuals [16–19]. However, there is a lack of reports identifying and validating direct miRNA targets, which is needed to better understand the involvement of miRNAs in processes relevant for respiratory disease pathogenesis.

In this study, we hypothesized that miRNAs are involved in the regulation of epithelial barrier dysfunction seen in chronic respiratory diseases, such as asthma. We therefore aimed to identify miRNAs involved in airway epithelial barrier damage caused by inflammatory stimuli, including IL-4 + IL-13, IL-17A + TNFα, IL-6 + sIL-6R, and TGFβ, representative of different asthma endotypes. To answer this question, we used air–liquid interface (ALI) cultures of human bronchial epithelial cells, RNA sequencing, qPCR, miRNA pull-down, miRNA mimics, and antagomirs. Moreover, miRNAs of interest were evaluated in airway samples from subjects with asthma.

## Methods

### Air–liquid interface cultures of primary bronchial epithelial cells

Primary normal human bronchial epithelial cells from five donors (HBECs; Lonza, Basel, Switzerland) were expanded to passage 2, then seeded onto 0.4 µm Corning® HTS Transwell®−24 well permeable polyester supports (Sigma-Aldrich) and differentiated at Air–Liquid Interface (ALI) using the PneumaCult™ media system (STEMCELL Technologies) according to the manufacturer’s protocol [20] for four weeks until fully differentiated. Before the ALI cultures were used for experiments, the cultures were assessed visually with a light microscope to confirm cilia movement and the presence of mucus. Furthermore, it was ensured that no leakage of media was present on the apical side.

### Barrier integrity assessment

Fluorescein isothiocyanate (FITC)-Dextran (FD4, Sigma-Aldrich) was used as in Horndahl et al. [20]. Briefly, FITC-Dextran diluted to 0.5–1 mg/ml in PneumaCult™ media was apically added to cells (100–200 µl) and allowed to pass through to the basolateral compartment under normal growth conditions for 18 h. Thereafter, supernatant samples were collected in triplicates from the basolateral compartment and fluorescence (Ex: 485 nm, Em: 520 nm) was measured on a PHERAstar Plus plate reader (BMG Labtech). The fluorescence signal was normalized to that from unstimulated/untreated control cells within each experiment (n = 3–7).

### Cytokine stimulation

Cells grown on transwells were stimulated basolaterally with human recombinant cytokines diluted in PneumaCult™-ALI medium (as previously described [9, 21] and see Supplementary Table 1 for details) and re-applied daily with length of stimulation as stated in the results (24–96 h).

### Cytotoxicity assay

To assess acute cytotoxicity, the release of lactate dehydrogenase (LDH) into conditioned media was measured using the CyQUANT LDH Cytotoxicity Assay (Thermo Fisher Scientific). Equal amounts of sample and reaction mixture were mixed in a clear flat-bottom 384-well plate (Greiner) and incubated in the dark at room temperature for 30 min before adding the stop solution. Absorbance values at 490 and 680 nm were obtained using a Spectramax reader (Molecular Devices), the difference was determined and used to calculate the fold change of samples from stimulated cells versus unstimulated cells.

### RNA isolation and next-generation sequencing

RNA was isolated from cells lysed in QIAzol Lysis Reagent (QIAGEN) using RNeasy, miRNeasy, and RNeasy MinElute Cleanup Kits (QIAGEN) following the manufacturer’s instructions. For sequencing, total RNA was separated, as described in the protocol in Appendix A of the miRNeasy kit, into two fractions containing fragments longer and shorter than 200 nucleotides, respectively. Therein, the miRNA-enriched fraction (< 200 nucleotides) was purified from the flow-through from the spin column using the RNeasy MinElute Cleanup Kit and the longer nucleotides were bound to and purified from the spin column.

Next-generation sequencing (NGS) of the > 200 nucleotide RNA fraction has been described previously, and parts of the data from the IL-4 + IL-13, IL-17A + tumor necrosis factor alpha (TNFα), and IL-6 trans-signaling stimulated cells have been previously published [9, 21]. Briefly, the integrity of the RNA was measured using a Bioanalyzer 2100 instrument (Agilent). The average RIN value was 9.5 (range 8.3–10) for all the samples included. RNA was converted to libraries using the TruSeq Stranded mRNA kit (Illumina) and sequenced on an Illumina NextSeq500 platform in 2 × 76 cycles on a High Output flow cell. Differential gene expression was assessed with DESeq2 [22], using raw counts as input. Differential expression was considered significant when q(p-adj) < 0.05 following Benjamini–Hochberg multiple correction.

The < 200 nucleotide RNA fraction was assessed by Fragment Analyzer (Agilent, standard sensitivity RNA 15-nt), and all samples included showed a distinct peak at 140 nt. NGS libraries were generated using SMARTer smRNA-Seq Kit for Illumina (Clontech) with size selection using Agencourt AMPure XP Beads. The quality of the resulting libraries was analyzed by Fragment Analyzer standard sensitivity NGS kits (Agilent), and libraries were pooled in an equimolar ratio. Sequencing was performed on an Illumina NextSeq500 platform with 60 cycles on a High Output flow cell. Differential miRNA expression was assessed with DESeq2. However, since fewer miRNAs than protein-coding genes had q < 0.05, miRNAs with p < 0.05 were considered for further analysis.

### qPCR on RNA from ALI cultured bronchial epithelial cells

For quantitative PCR (qPCR), total RNA isolated from cells as described above in "RNA isolation and next-generation sequencing" section (*n* = 2–5) was converted into cDNA using the High-Capacity cDNA Reverse Transcription Kit (Applied Biosystems) for mRNA analysis and the TaqMan® Advanced miRNA cDNA Synthesis Kit (Applied Biosystems) for miRNA analysis. qPCR was run using the comparative Ct method on a QuantStudio 7 Flex Real-Time PCR system (Thermo Fisher Scientific) using TaqMan™ Fast Advanced Master Mix (Thermo Fisher Scientific). Gene expression was determined with probes from TaqMan Real-Time PCR Assays (Thermo Fisher Scientific) and normalized against the housekeeping gene ACTB as in [21]. For miRNA, equal amounts of RNA were converted and analyzed, and differential expression was assessed using TaqMan Advanced miRNA Assays (Thermo Fisher Scientific). Normalization was performed against the geometric mean of miR-28-3p and miR-186-5p, which showed low variation in expression across stimuli when using both NGS and qPCR (*n* = 3–5). For details about the reagents, see Supplementary Table 2.

### Pull-down experiments

To analyze miRNA-mRNA interactions, pull-down experiments were performed using a protocol previously established in our lab [23, 24]. Cell lysates from untreated HBEC-ALI cultures were incubated with biotin-tagged miRNA mimics for hsa-miR-146a-3p, hsa-miR-363-3p, or a negative control mimic (all from QIAGEN). See Supplementary Table 3 for details about the reagents. Magnetic streptavidin beads (Thermo Fisher Scientific) were then used to pull down the mimic-mRNA duplexes from the lysates. RNA was isolated using RNeasy kits as described above, and the presence of target mRNAs of interest were analyzed by qPCR, with relative quantification calculated against the negative control mimic (*n* = 2–3).

### Liquid-covered culture and transfection of miRNA mimic

Due to the difficulty in transfecting HBEC-ALI, an alternative cell model was developed where primary cells in passage 3 were seeded on transwells as described above, but instead of being exposed to air, the cells were kept covered with media apically. The media was then changed on both apical and basolateral sides every other day. Approximately 6–7 days after seeding, this liquid-covered culture formed a tight barrier, which could be disrupted through daily addition of IL-17A + TNFα (data not shown).

Cells were transfected according to the manufacturers’ instructions 6 days after seeding. Complexes consisting of Lipofectamine RNAiMAX (Life Technologies) and miRNA mimics (QIAGEN) diluted in OptiMEM (Life Technologies) were added to the apical side of the cells, whereas the basolateral media remained the same. This was repeated for every media change (every 2–3 days) until day 12 after seeding. The cells were then lysed in QIAzol and kept at −80 °C until further analysis (*n* = 3). For details about the reagents, see Supplementary Table 3.

### Administration of miRNA antagomirs to cytokine-stimulated ALI cultures

HBECs were differentiated at ALI as described above and apically washed by incubation with 200 µl PBS for 2 h to remove accumulated mucus. After this, antagomirs (miR-363-3p or Negative control A, both Power Inhibitors from QIAGEN) diluted in PBS or a PBS only control (vehicle) were added to the apical (25 µl) and basolateral side of cells to a final concentration of 100 nM and incubated for 6 h before cytokines/vehicle were added to the basolateral side. Media with/without cytokines and antagomirs, and PBS with/without antagomirs were re-applied daily to the basolateral and apical side, respectively. The cells were then lysed in QIAzol and kept at −80 °C until further analysis. For details about the reagents, see Supplementary Table 3.

### Study participants

Study participants were either invited from the random epidemiological cohort West Sweden Asthma Study (WSAS) or recruited via advertisement [25]. All participants completed a self-administered questionnaire, a structured clinical interview, and a clinical examination. All individuals with asthma had physician-diagnosed mild to moderate asthma and were on inhaled corticosteroid treatment. Atopy was determined by positive skin prick test and/or specific serum IgE levels. Control subjects did not report asthma symptoms. Spirometry, differential cell count, and detailed patient history were included for all subjects. This study was conducted in accordance with the Declaration of Helsinki and was approved by the Gothenburg Regional Ethical Committee (no.228–14), and written informed consent was obtained from all study participants.

### Bronchial lavage fluid sampling

Bronchial lavage (BL) was performed during bronchoscopy at the Lung Diagnostic facility at Sahlgrenska University Hospital, Gothenburg, Sweden. BL fluid was obtained by thrice instilling and retrieving 20 ml sterile pyrogen-free PBS. The second and third washes were pooled and filtered using a 70 µm cell strainer. The BL sample was centrifuged to separate cells from fluid, and one tablet of proteinase inhibitor cocktail (cOmplete mini EDTA-free; Roche Diagnostics GmbH, Mannheim, Germany) was added per 10 ml aliquot of cell-free BL fluid before storage at −80 °C until further analysis. Stained cytospin slides were prepared from the cells for analysis of the immune cell composition in BL by differential cell count.

### RNA isolation and miRNA expression analysis of BL samples using qPCR

Total RNA was isolated from 600 µl sterile filtered cell-free BL fluid using miRNeasy microKit (QIAGEN, Hilden, Germany). The procedure was carried out according to the manufacturer’s instructions, with the exception that 700 µl QIAzol was used per 150 µl sample aliquot and Phasemaker™ tubes (Invitrogen by Thermo Fisher Scientific, Life Technologies Corp. Carlsbad, CA, USA) were utilized in the phase separation stage as described in the manufacturer’s user guide.

cDNA was reverse transcribed using miRCURY LNA RT Kit (QIAGEN, Hilden, Germany) and a fixed volume of 4 µl total RNA per cDNA reaction. qPCR was performed on a CFX96 Touch Real-Time PCR Detection System (Bio-Rad Laboratories, Hercules, CA, USA) using cDNA diluted 1:40 and human LNA primers for miR-363-3p (QIAGEN) and miR-103a-3p (QIAGEN), and miRCURY LNA SYBR Green PCR kit (QIAGEN). The relative expression (fold change) of miR-363 was calculated using the 2^−ΔΔCt^ method, where miR-363-3p was normalised to the reference miRNA, miR-103a-3p, and the mean value of healthy control subjects. For details about the reagents, see Supplementary Table 4.

### Bioinformatics and data analysis

The databases miRDB [26], TargetScan [27], and miRWalk [28] were used for prediction of putative miRNA targets as well as for the prediction of the binding sites and strength between a miRNA and a target mRNA (accessed January 2020). This was done using a script written in R Studio along with a list of barrier-related genes compiled from literature [10, 12, 29, 30].

GraphPad Prism v.8 (GraphPad Software, La Jolla, CA, USA) or R Studio was used for data analysis, including statistical calculations as stated in the results.

The Database for Annotation, Visualization and Integrated Discovery (DAVID; https://davidbioinformatics.nih.gov/, accessed December 2025) was used to determine the most enriched biological functions associated with upregulated mRNA in the NGS analysis [31, 32].

For the study participants, the clinical data are presented as median (Q1–Q3) or n (%). The statistical package for social sciences (SPSS) for Windows version 29 (IBM Corp., Armonk, NY USA) and GraphPad Prism for Windows version 10.3 (GraphPad Software, Boston, Massachusetts USA) were used for statistical analysis. The Mann–Whitney U test was used for comparisons between groups, and correlations were performed using Spearman’s Rank correlation test.

## Results

### Th17 inflammation causes airway epithelial barrier dysfunction

**Fig. 1:**
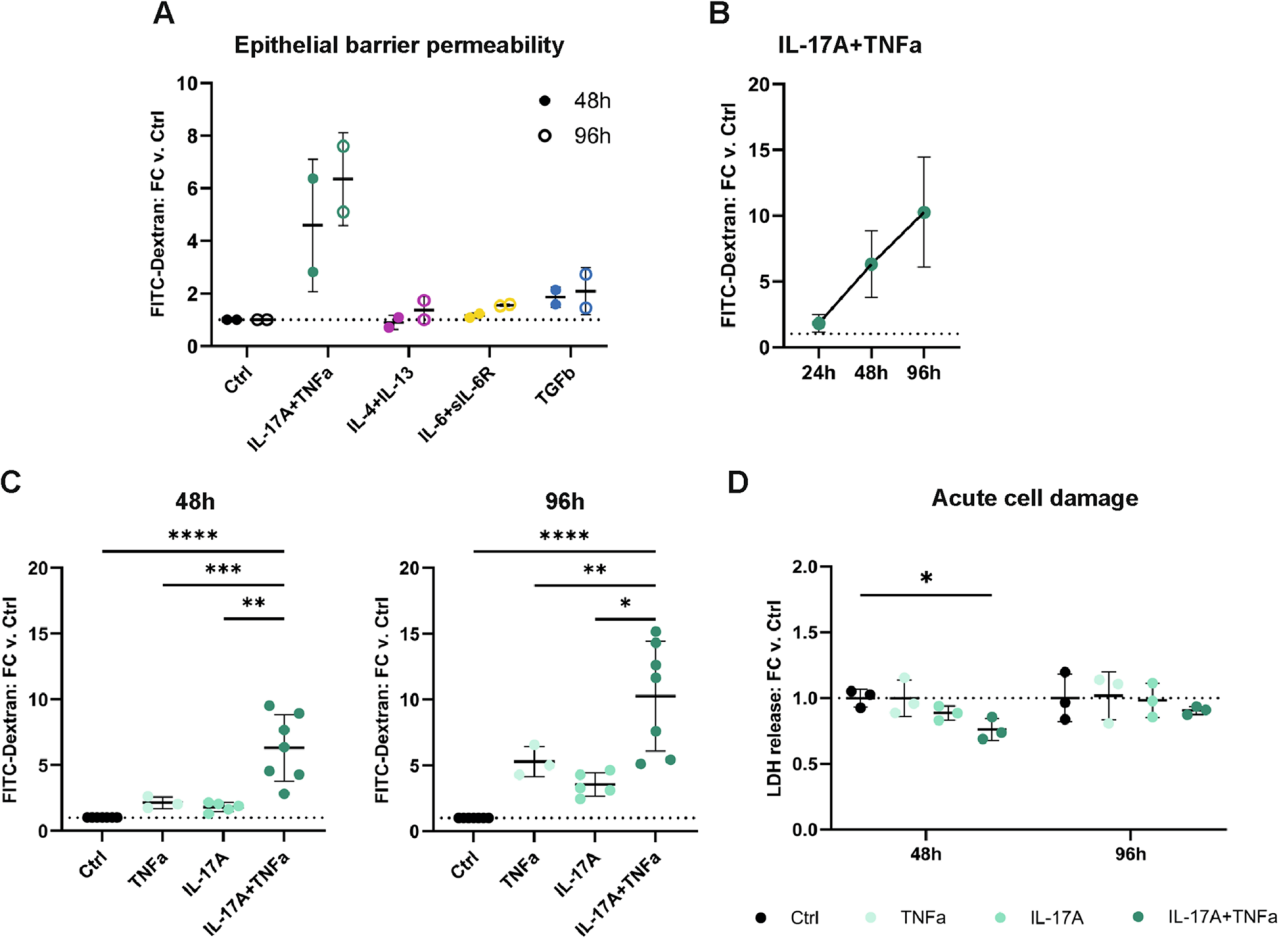
IL-17A and TNFα have additive disruptive effects on the integrity of the airway epithelial barrier. **A** Human primary bronchial epithelial cells cultured at air–liquid interface (HBEC-ALI) were stimulated basolaterally with cytokines representative of different inflammatory endotypes, and the effect on the epithelial barrier was assessed by paracellular flux of FITC-labelled dextran (*n* = 2 donors). **B** Since a disruptive effect was observed after IL-17A + TNFα stimulation, the effect was validated over time in several donors (*n* = 3–7, from 5 donors across 3 experiments). **C** The effect of IL-17A and TNFα separately or combined on the epithelial barrier was also further determined (*n* = 3–7, from 5 donors across 3 experiments). **D** The level of lactate dehydrogenase released into the basolateral media was measured at 48 h and 96 h to determine if IL-17A and/or TNFα caused any cell damage. The data is presented as mean ± SD. Dotted lines on the y-axis indicate fold change = 1. Statistical calculations are Ordinary ANOVA with Tukey´s test for C-D. * *p*-value < 0.05, ** *p*-value < 0.01, *** *p*-value < 0.001, **** *p*-value < 0.0001. Abbreviations: Ctrl, control; FC, fold change; h, hours

An airway epithelium in vitro cell model (primary HBEC-ALI) was used to determine the effects of different inflammatory milieus on the airway epithelium barrier function. To model different inflammatory environments, disease-relevant cytokines known or predicted to contribute to airway inflammation were used to stimulate the cells: IL-4 + IL-13 for T2 inflammation [8, 33], IL-17A + TNFα for Th17 inflammation [8, 33], IL-6 + soluble IL-6R for IL-6 trans-signaling [9, 34], and transforming growth factor beta (TGFβ) which is associated with remodeling and T regulatory cells and, therefore, is context-dependent [35]. FITC-Dextran was used to assess epithelial barrier integrity upon cytokine stimulation. IL-17A + TNFα induced the strongest increase in epithelial barrier permeability, and it increased over time with the strongest effect at 96 h (Fig. 1A-B). This was a synergistic effect dependent on the combination of IL-17A and TNFα, as it was not observed when the cells were treated with the individual cytokines (Fig. 1C). The impaired barrier function was not driven by acute cell damage, as there was no increase in lactate dehydrogenase released to the basolateral medium from cytokine-stimulated cells compared to non-stimulated controls (Fig. 1D). Together, this suggests that Th17-mimicking inflammatory conditions induce airway barrier disruption that is not due to cell death.

### Specific gene expressions are induced upon cytokine stimulation

**Fig. 2:**
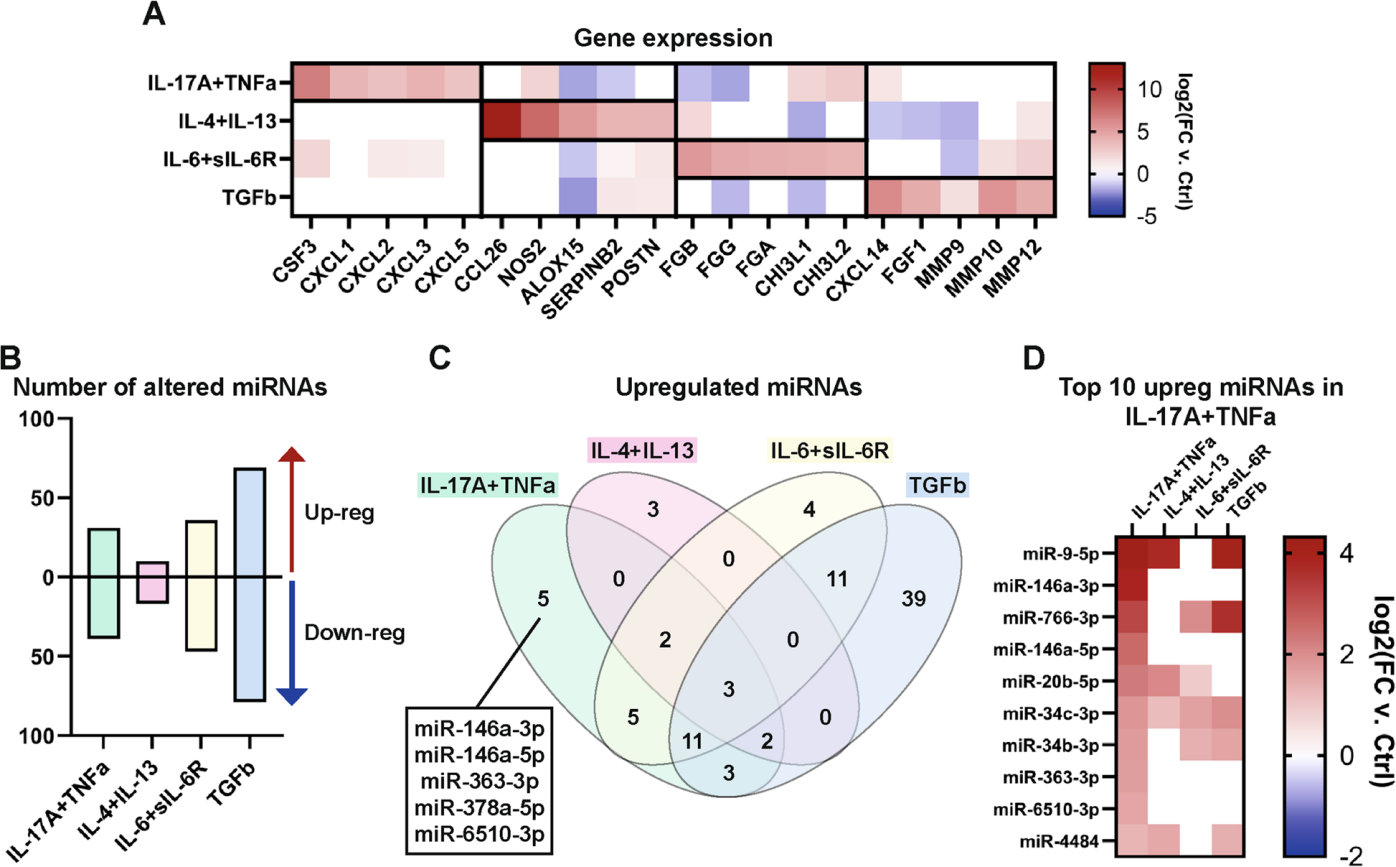
IL-17A + TNFα alters miRNA expression in airway epithelial cells. NGS was used to identify mRNAs and miRNAs in HBEC-ALI influenced by cytokines representative of different inflammatory endotypes. **A** The expression of a selection of genes that has previously been identified as altered in airway epithelial cells upon cytokine stimulation. **B** The number of significantly upregulated and downregulated miRNAs after cytokine stimulation compared to untreated cells. **C** The number of uniquely and mutually upregulated miRNAs in the four cytokine stimulations. **D** The top 10 most enriched significant miRNAs after IL-17A + TNFα stimulation and their expression after the other stimuli. Red, upregulated, and blue, downregulated. Abbreviations: Ctrl, control; FC, fold change

To determine the response to the cytokine stimulations, gene expression was analyzed by NGS in the cytokine-stimulated HBEC-ALI. To visualize the variation after stimulation, we used principal component analysis (PCA). Component 1, representing 28% of the variance, distinguished the TGFβ stimulation from the other stimulations and the control cells (Supplementary Fig. 1A). Component 2, representing 19% of the variance, separated IL-6 + sIL-6R and IL-17A + TNFα from IL-4 + IL-13, TGFβ stimulation, and control cells. Next, we determined the most enriched biological functions associated with the upregulated mRNA in each stimulation. From an analysis of the induced protein-coding genes, it was clear that the cells had responded differently to the cytokine stimulations (Supplementary Fig. 1B-E). Both IL-17A + TNFα and IL-4 + IL-13 induced genes associated with inflammatory response and innate immune responses. Furthermore, IL-17A + TNFα induced genes associated with neutrophil chemotaxis, while IL-4 + IL-13 induced genes associated with o-glycan processing, which is related to mucin production (Supplementary Fig. 1B-C). On the other hand, genes associated with mitochondria were upregulated in IL-6 + sIL-6R, and genes associated with degrading extracellular matrix were associated with TGFβ (Supplementary Fig. 1D-E). Next, we focused on genes that have been previously shown to be altered upon cytokine stimulation [33, 36–40] (Fig. 2A). For IL-17A + TNFα, the neutrophil-stimulating protein, granulocyte colony-stimulating factor (*CSF3*), and neutrophil chemoattractants (chemokine [C-X-C motif] ligand 1 [*CXCL1*], *CXCL2, CXCL3*, and *CXCL5)* were upregulated. For IL-4 + IL-13, some of the most upregulated genes were the eosinophil-attracting chemokine eotaxin-3 (*CCL26*), inducible nitric oxide synthase (*NOS2*), *ALOX15*, plasminogen activator inhibitor-2 (*SERPINB2*), and periostin (*POSTN*). *NOS2*, *POSTN*, and *SERPINB2* have all been identified as markers for T2 inflammation [41–43]. IL-6 is a main regulator of fibrinogen synthesis, and after IL-6 + soluble IL-6 receptor (sIL-6R) stimulation, fibrinogens (*FGA*, *FGB*, *FGG*) were among the most upregulated genes. Additionally, chitinase-3-like protein 1 and 2 (*CHI3L1* and *CHI3L2*) increased after IL-6 + sIL-6R stimulation. *CHI3L1* has been associated with T2-low inflammation in asthmatics, and a polymorphism in the *CHI3L1* gene is associated with risk of asthma [44, 45]. TGFβ stimulation also elicited a markedly distinct response in the HBEC-ALI compared to the other stimulations. Several matrix metalloproteinases (*MMP9*, *MMP10*, *MMP12*), as well as *FGF1* and *CXCL14,* were upregulated. MMPs are enzymes that degrade extracellular matrix proteins, and CXCL14 is a chemoattractant for immune cells and has been associated with asthma [46]. Lastly, FGF1 increases the proliferation of lung cells and is increased in epithelial cells in chronic obstructive pulmonary disease (COPD) [47]. This aligns with TGFβ being involved in cell growth, tissue remodeling, fibrosis, and immune regulation. Together, this shows that the gene responses in HBEC-ALI reflect the different immune responses that the cytokines mimic, validating the relevance of the model.

### Th17 inflammation increases the expression of miRNAs with putative epithelial barrier-related target genes

Impaired epithelial barrier function is usually related to a decreased level, improper localization, or altered function of proteins involved in forming the different types of junctions between cells or between cells and the basement membrane [11, 13, 14]. To identify miRNA-mRNA interactions that could lead to a decreased expression of barrier-related genes, the expression of miRNAs in cytokine-stimulated HBEC-ALI cultures was also determined by RNA sequencing.

In total, 434 miRNAs were identified by miRNA NGS analysis. First, PCA was utilized to visualize the variation after stimulation. Component 1, representing 45% of the variance, distinguished the TGFβ stimulation from the other stimulations and the control cells again (Supplementary Fig. 2A). Component 2, representing 16% of the variance, separated IL-6 + sIL-6R and IL-17A + TNFα from IL-4 + IL-13, TGFβ stimulation, and non-stimulated cells again. This together with the mRNA analysis, suggests that TGFβ and IL-6 + sIL-6R altered the gene and miRNA expression the most in HBEC-ALI, while IL-4 + IL-13 were the most similar to the non-stimulated cells. Of the 434 miRNAs identified with miRNA NGS, 70, 27, 83, and 148 miRNAs were significantly differentially expressed in IL-17A + TNFα, IL-4 + IL-13, IL-6 + sIL-6R, and TGFβ compared to untreated control, respectively (Fig. 2B). As we found IL-17A + TNFα treatment to have the most disruptive effects on the epithelial barrier in vitro*,* we focused our analysis on this cytokine stimulation. Of the 70 miRNAs altered by IL-17A + TNFα stimulation, 31 and 39 miRNAs were significantly upregulated or downregulated, respectively (Fig. 2B). Compared to the other stimulations, thirteen (five upregulated and eight downregulated) miRNAs were uniquely altered by IL-17A + TNFα stimulation (Fig. 2C and Supplementary Fig. 2B). Four of the five uniquely upregulated miRNAs (miR-146a-3p, miR-146a-5p, miR-363-3p, and miR-6510-3p) were also among the 10 most upregulated miRNAs after IL-17A + TNFα stimulation (Fig. 2D).

**Fig. 3:**
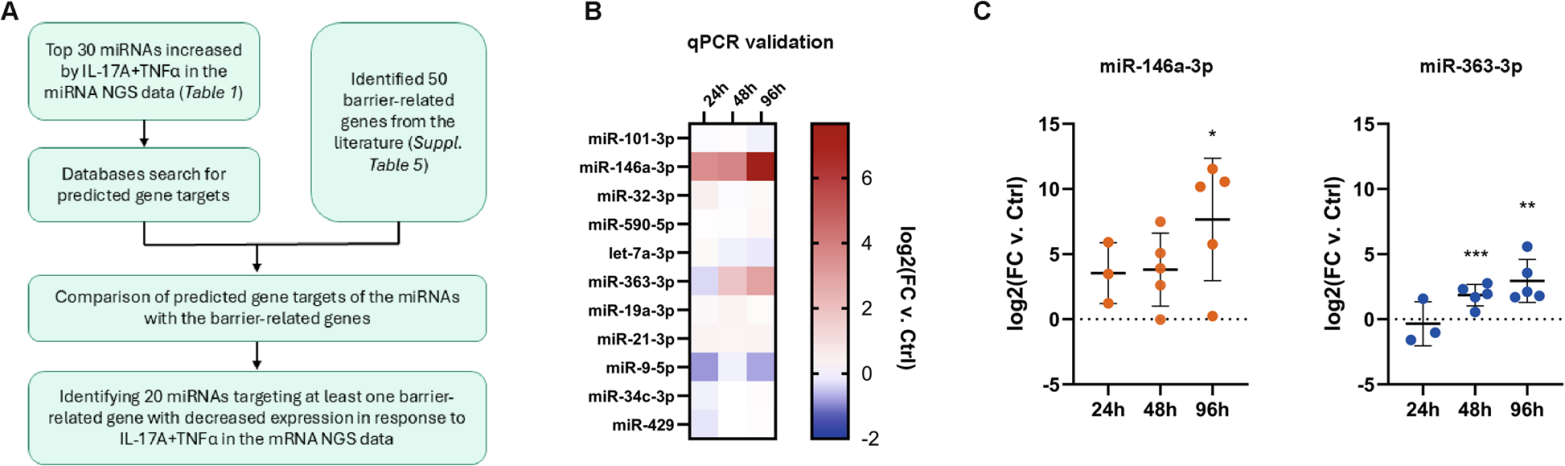
IL-17A + TNFα causes increased expression of several miRNAs predicted to target barrier-related genes. NGS was used to identify miRNAs and mRNAs affected by IL-17A + TNFα in HBEC-ALI. **A** Schematic overview of the bioinformatic analysis where the 30 most increased miRNAs (Table 1) were assessed for possible targeting of barrier-related genes (Supplementary Table 5) that were decreased in the mRNA NGS data. This resulted in 20 such miRNAs. **B** Eleven of these twenty miRNAs were chosen for validation by qPCR, and out of these, miR-146a-3p and miR-363-3p were increased across multiple donors of HBEC-ALI stimulated with IL-17A + TNFα (data presented as mean values across donors, *n* = 3–5). Red, upregulated, and blue, downregulated. **C** The individual values from the qPCR analysis of miR-146a-3p and miR-363-3p expression. The data is presented as mean ± SD. Dotted lines on the y-axis indicate log2 fold change = 0. Statistical calculations are Ordinary ANOVA with Tukey´s test. * *p*-value < 0.05, ** *p*-value < 0.01, *** *p*-value < 0.001. Abbreviations: Ctrl, control; FC, fold change; h, hours

Next, we identified 50 barrier-related genes from the literature [10, 12, 29, 30], of which many had decreased expression in the mRNA sequencing data after IL-17A + TNFα stimulation (Supplementary Table 5). TargetScan, miRDB, and miRWalk were then used to identify mRNA targets for the 30 most increased miRNAs after IL-17A + TNFα stimulation (Table 1 and Fig. 3A**)**. Comparing this list of putative target mRNAs to the list of barrier-related genes (Supplementary Table 5), it was found that twenty of the top thirty miRNAs induced by IL-17A + TNFα were predicted to target at least one barrier-related gene exhibiting decreased expression in response to IL-17A + TNFα stimulation. Eleven miRNAs were chosen for validation by qPCR based on having either unique or multiple predicted target genes (Fig. 3B). Of these, miR-146a-3p and miR-363-3p were shown to be consistently upregulated after stimulation with IL-17A + TNFα in multiple donors (Fig. 3C). The increased expression was strongest at the later timepoints (48 h and 96 h), coinciding with the strongest barrier effect (Fig. 1B).

**Table 1:** The top 30 most increased miRNAs in HBEC-ALI stimulated basolaterally with IL-17A + TNFα for 24 h compared to non-stimulated control (*n* = 1)

miRNA	log2(FC)	*p*-value
hsa-miR-9-5p	4.31	0.01369
hsa-miR-146a-3p	3.85	9.39E-07
hsa-miR-766-3p	3.20	0.04462
hsa-miR-146a-5p	2.59	2.74E-10
hsa-miR-20b-5p	2.28	0.00022
hsa-miR-34c-3p	1.85	0.00182
hsa-miR-34b-3p	1.74	0.00626
hsa-miR-363-3p	1.69	0.01652
hsa-miR-6510-3p	1.59	0.04146
hsa-miR-4484	1.32	0.04049
hsa-miR-190b	1.23	0.03295
hsa-miR-141-5p	1.03	0.00385
hsa-miR-33a-3p	0.98	0.00904
hsa-miR-19b-3p	0.91	0.00263
hsa-miR-32-3p	0.89	0.00545
hsa-miR-19a-3p	0.87	0.00244
hsa-miR-590-5p	0.85	0.00863
hsa-miR-4284	0.79	0.02321
hsa-miR-339-3p	0.79	0.01517
hsa-miR-429	0.78	0.00031
hsa-miR-378a-5p	0.75	0.03104
hsa-miR-99a-5p	0.67	0.01365
hsa-let-7c-5p	0.63	0.00317
hsa-miR-200c-5p	0.61	0.01292
hsa-miR-200a-5p	0.60	0.00403
hsa-miR-101-3p	0.58	0.01904
hsa-let-7a-3p	0.56	0.04632
hsa-miR-21-3p	0.49	0.00278
hsa-miR-31-3p	0.48	0.03160
hsa-let-7 g-5p	0.41	0.01310

FC: fold change, *p*-value: standard *p*-value, p.adj: *p*-value after multiple comparison (FDR)

**Fig. 4:**
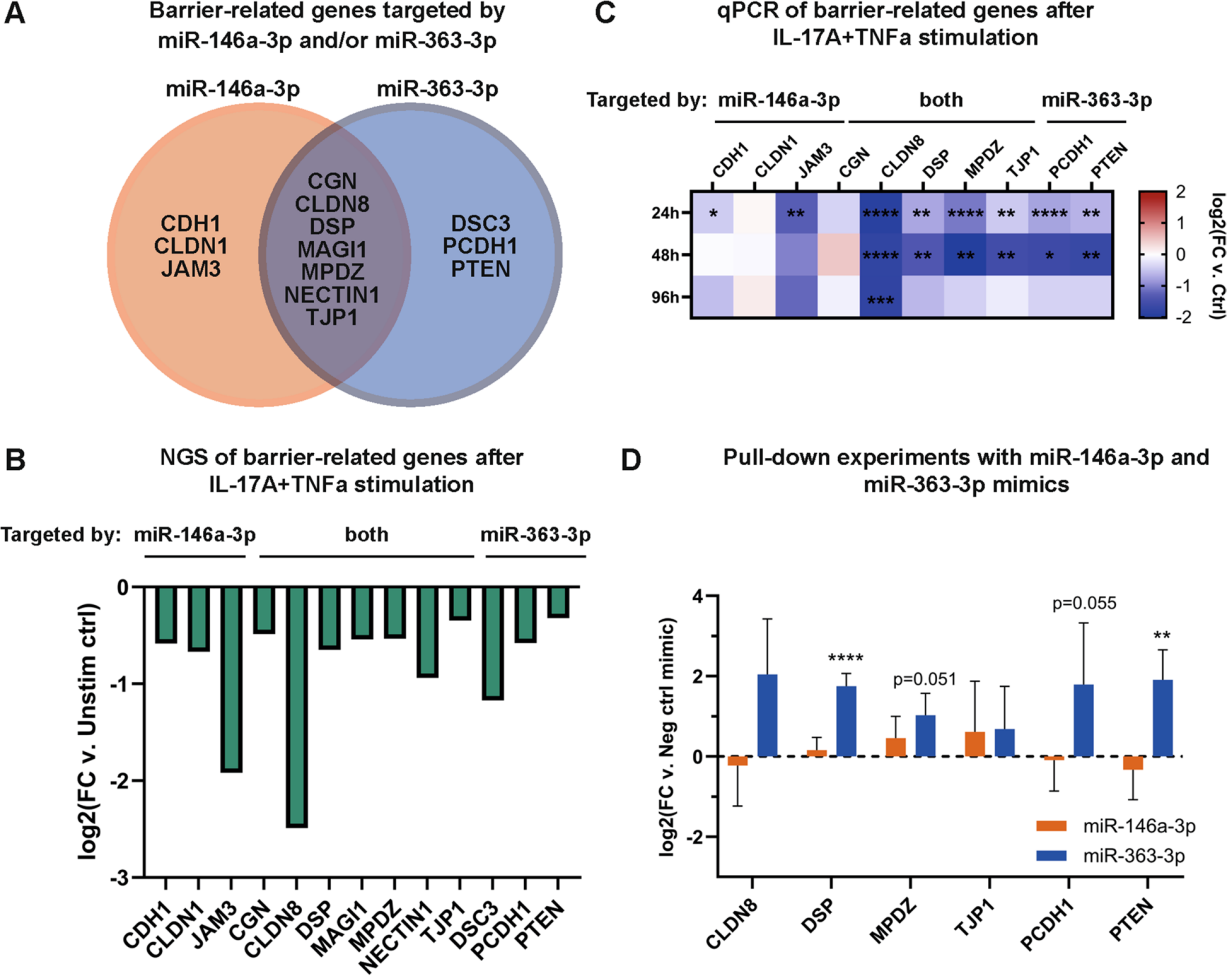
Barrier-related genes predicted to be targeted by miR-146a-3p and miR-363-3p are decreased in response to IL-17A + TNFα stimulation. **A** Predicted barrier-related targets of miR-146a-3p and miR-363-3p according to the databases miRDB, miRWalk, and TargetScan. **B** The expression of the predicted barrier-related target genes for miR-146a-3p and miR-363-3p in the NGS analysis after IL-17A + TNFα. **C** Ten targets were chosen for validation by qPCR, and several of the predicted targets were decreased across multiple donors after stimulation with IL-17A + TNFα. Data presented as mean values across donors, *n* = 2–5. Statistical calculations using Ordinary ANOVA with Tukey´s test. Red, upregulated, and blue, downregulated. **D** To evaluate if miR-363-3p and miR-146a-3p interacted with the predicted barrier-related mRNAs, pull-down experiments using miRNA mimics on cell lysates from untreated HBEC-ALI cultures, followed by qPCR, were used. The data is presented as mean ± SD, *n* = 3–4 donors. Statistical calculations are Ordinary ANOVA with Dunnett´s test, with comparison to negative control mimic. * *p*-value < 0.05, ** *p*-value < 0.01, *** *p*-value < 0.001, **** *p*-value < 0.0001. Abbreviations: CDH1, cadherin-1; CGN, cingulin; CLDN1, claudin-1; CLDN8, claudin-8; DSC3, desmocollin-3; DSP, desmoplakin; FC, fold change; JAM3, junctional adhesion molecule C; MAGI1, membrane-associated guanylate kinase, WW and PDZ domain-containing protein 1; MPDZ, multiple PDZ domain protein; NECTIN1, nectin-1; PCDH1, protocadherin-1; PTEN, phosphatidylinositol 3,4,5-trisphosphate 3-phosphatase and dual-specificity protein phosphatase PTEN; TJP1, tight junction protein ZO-1

From the database analyses, miR-146a-3p and miR-363-3p were predicted to target a total of 13 barrier-related genes (Fig. 4A), with all of them being downregulated in the NGS data after IL-17A + TNFα stimulation (Fig. 4B). Importantly, seven of these (claudin-8 [*CLDN8*], desmoplakin [*DSP*], junctional adhesion molecule C [*JAM3*], multiple PDZ domain protein [*MPDZ*], protocadherin-1 [*PCDH1*], phosphatase and tensin homolog [*PTEN*]*,* and tight junction protein ZO-1 [*TJP1*]) were validated by qPCR to be significantly decreased by IL-17A + TNFα (Fig. 4C). These results suggest that Th17 inflammation causes dysfunctional epithelial barrier function through the induction of miRNAs that decrease the levels of mRNA transcripts encoding barrier proteins.

### miR-363-3p interacts with mRNA transcripts of epithelial barrier components

To confirm interaction between the two miRNAs and their putative targets, pull-down experiments were performed with lysates from fully differentiated HBEC-ALI cultures. Analysis by qPCR focused on the 6 most significantly affected genes from Fig. 4C and confirmed significant interactions between miR-363-3p and mRNA transcripts of the epithelial barrier genes *DSP* and *PTEN* and there was a trend towards interaction with the mRNAs for *CLDN8*, *MPDZ*, *PCDH1*, and *TJP1*, although with large donor variability (Fig. 4D). Only *TJP1* showed a trend towards interaction with miR-146a-3p, however, there was again a large variation in binding levels among donors (Fig. 4D). Thus, the observed reduced levels of barrier-related gene transcripts in Fig. 4B-C may be mediated by miR-363-3p.

### miR-363-3p mimic and miR-363-3p antagomir affect the expression of epithelial barrier-related genes

**Fig. 5:**
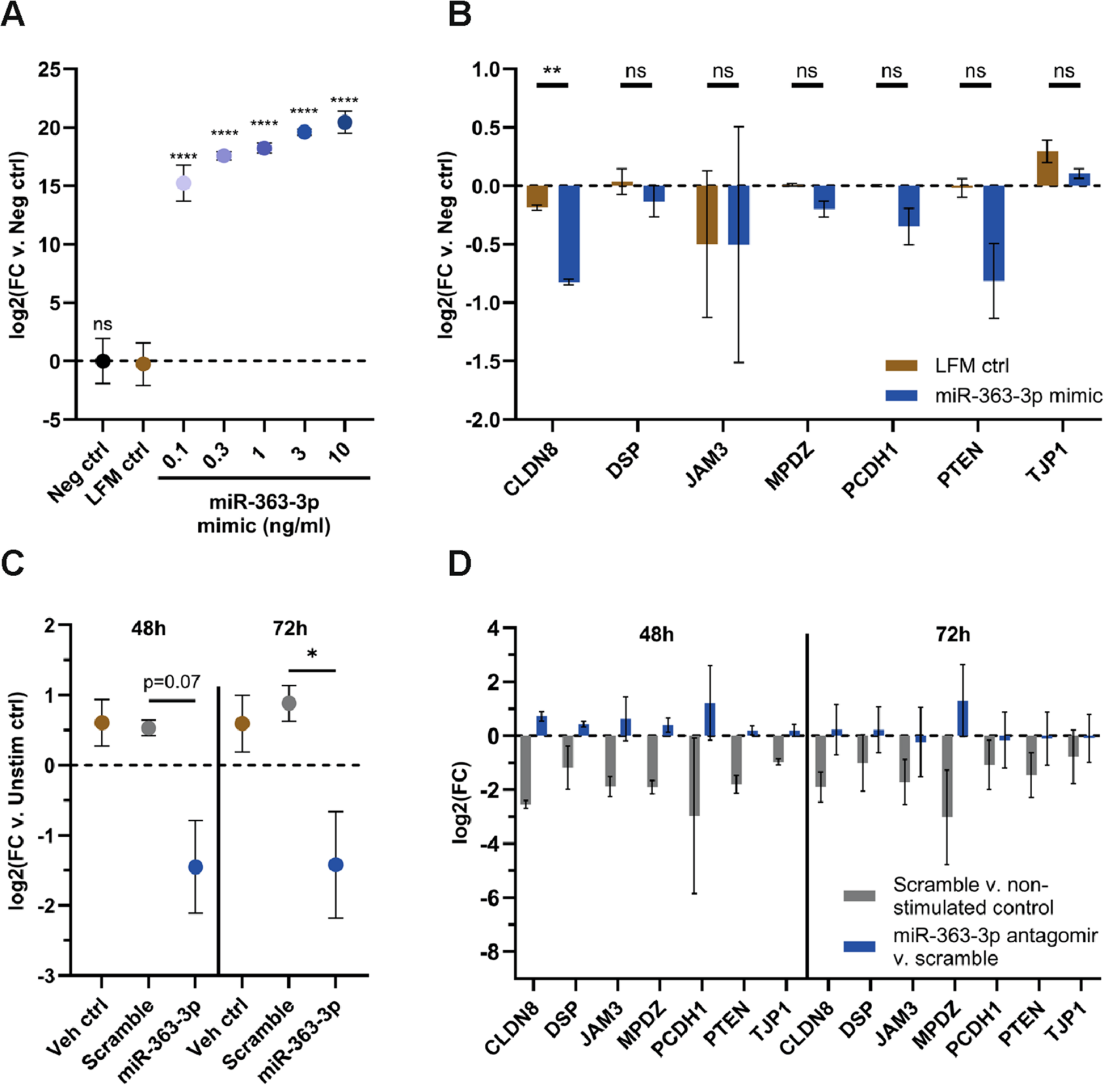
miR-363-3p mimic and antagomir affect the expression levels of barrier-related genes in primary airway epithelial cell cultures.** A** HBEC-ALI cultured in a submerged state were incubated with Lipofectamine-mimic complexes for six days, with re-application every other day. Analysis by qPCR showed a dose-dependent increase of the transfected miR-363-3p. Data presented as mean ± SD, *n* = 1 donor, 2–3 replicates. **B** qPCR analysis showed decreased levels for some of the target genes after the addition of 3 ng/ml miR-363-3p mimic compared to control (*n* = 1 donor, 2–3 replicates). **C** Antagomirs (100 nM) in PBS were administered apically and basolaterally to normal HBEC-ALI cultures stimulated with IL-17A + TNFα. Cytokines and antagomirs were reapplied daily. Analysis by qPCR showed decreased levels of miR-363-3p at both 48 and 72 h. Data presented as mean ± SD, *n* = 3 donors. **D** Analysis by qPCR showed partial restoration of decreased expression of some of the barrier-related genes. Data presented as mean ± SD, *n* = 3 donors. Statistical calculations using Ordinary ANOVA against LFM ctrl with Dunnett´s test for A and Ordinary ANOVA against Scramble with Bonferroni´s test for C. Multiple t-tests adjusted with Benjamini and Hochberg for B and D. ns = non-significant, * *p*-value < 0.05, ** *p*-value < 0.01, **** *p*-value < 0.0001. Abbreviations: CLDN8, claudin-8; Ctrl, control; DSP, desmoplakin; FC, fold change; h, hours; JAM3, junctional adhesion molecule C; LFM, lipofectamine; MPDZ, multiple PDZ domain protein; PCDH1, protocadherin-1; PTEN, phosphatidylinositol 3,4,5-trisphosphate 3-phosphatase and dual-specificity protein phosphatase PTEN; TJP1, tight junction protein ZO 1; Veh, vehicle

Fully differentiated HBEC-ALI cultures are difficult to transfect, thus an alternative liquid-covered cell culture model was developed (see Method "Liquid-covered culture and transfection of miRNA mimic" section). A miR-363-3p mimic was transfected into this model system, and a dose-dependent increase in the levels of the transfected mimic was confirmed using qPCR (Fig. 5A). Based on this, we chose to treat unstimulated cells with 3 ng/ml mimic and evaluated the expression of several of the barrier-related genes. The expression of *CLDN8* was significantly reduced by the miR-363-3p mimic, but non-significant reductions were also observed for *MPDZ*, *PCDH1*, and *PTEN* (Fig. 5B). In parallel, fully differentiated HBEC-ALI stimulated with IL-17A + TNFα were treated with an antagomir against miR-363-3p. Treatment with the antagomir strongly reduced the levels of miR-363-3p (Fig. 5C) and at the same time partly restored the expression of barrier-related genes, with the most pronounced effect at the 48 h timepoint (Fig. 5D). Together, this shows that the miR-363-3p mimic affects the expression of epithelial barrier-related genes, while a miR-363-3p antagomir ameliorates cytokine-triggered decrease in barrier gene expression. These findings, using the miR-363-3p mimic and antagomir, suggest that induction of miR-363-3p in response to treatment with IL-17A + TNFα may contribute to airway epithelial barrier dysfunction by targeting and reducing the expression of barrier-related genes.

### miR-363-3p expression in asthma

**Fig. 6:**
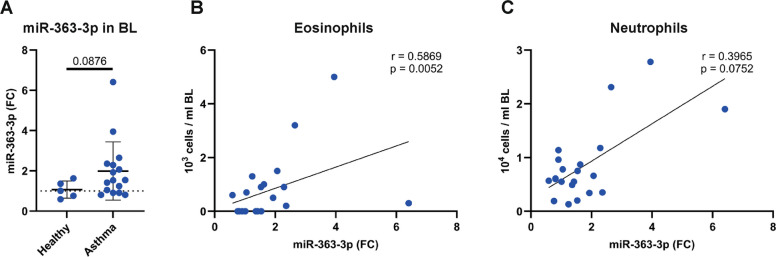
miR-363-3p expression in asthma.** A** The expression of miR-363-3p in bronchial lavage (BL) fluid from healthy controls and individuals with asthma analysed by qPCR. Values of miR-363-3p were normalized to miR-103-3p, and fold change (FC) was calculated relative to the mean value of all healthy controls. Data presented as mean ± SD, *n* = 5–16. **B**-**C** Associations between miR-363 expression and inflammatory cells in BL fluid. Eosinophil (**B**) and neutrophil (**C**) count in BL correlated to miR-363-3p relative expression in BL. Healthy controls and individuals with asthma were included in the analysis (*n* = 21). Statistical calculations using the Mann–Whitney U test for A and Spearman’s Rank correlation test for **B**-**C**. A linear line is shown for visualization in **B**-**C**

To further determine a possible role for miR-363-3p in asthma pathogenesis, we measured miR-363-3p expression in bronchial lavage (BL) samples from individuals with asthma and healthy individuals. In total, 16 individuals with asthma and 5 healthy control individuals participated in this study. The demographic and clinical characteristics of the participants showed that subjects with asthma had lower lung function as demonstrated by significantly lower FEV1% predicted and FEV1/FVC % (Table 2). Importantly, miR-363-3p showed a clear tendency of increased expression in the airways in individuals with asthma compared to healthy individuals (Fig. 6A). We further demonstrated a significant correlation between miR-363-3p expression and airway eosinophils (Fig. 6B) and a tendency towards correlation with airway neutrophils (Fig. 6C). Although individuals in our asthma cohort had mild to moderate asthma, our findings may have greater importance in a more severe asthma population with mixed eosinophilic-neutrophilic inflammation.

**Table 2:** Demographic and clinical characteristics of the study participants

	Healthy	Asthma	*p* value
Number, *n*	5	16	
Gender, M/F	3/2	11/5	
Age, years	29 (29–31)	50 (29–64)	0.208
Atopy, *n* (%)	0 (0%)	9 (56.3%)	
FEV_1_, % predicted	103.70 (100.00–114.10)	92.20 (82.30–100.20)	0.025
FEV_1_/FVC %	83.00 (83.00–87.00)	74.00 (69.50–78.50)	0.001
ICS treatment, *n* (%)	0 (0%)	16 (100%)	
Eosinophil count × 10^4^/ml BL	0.00 (0.00–0.06)	0.04 (0.00–0.11)	0.445
Neutrophil count × 10^4^/ml BL	0.55 (0.49–0.57)	0.71 (0.45–1.16)	0.208
Monocyte count × 10^4^/ml BL	0.29 (0.26–0.62)	0.20 (0.16–0.34)	0.275
Macrophage count × 10^4^/ml BL	8.35 (7.56–10.74)	6.35 (5.38–9.30)	0.354
Lymphocyte count × 10^4^/ml BL	0.70 (0.54–1.71)	0.56 (0.40–0.77)	0.313

Data are presented as median (Q1–Q3) or n (%). FEV_1_, forced expiratory volume in 1 s; FVC, forced vital capacity; ICS, inhaled corticosteroids; BL, bronchial lavage fluid

## Discussion

In this study, we demonstrate that stimulation with IL-17A and TNFα, mimicking Th17 inflammation in asthma, is disruptive to the airway epithelium in vitro, leading to increased barrier permeability and the downregulation of several barrier-related gene transcripts. Furthermore, IL-17A + TNFα increased the expression of miRNAs predicted to regulate the levels of epithelial barrier-related transcripts. miR-146a-3p and miR-363-3p were shown to be consistently increased by IL-17A + TNFα stimulation in multiple donors. In pull-down experiments, miR-363-3p was confirmed to bind to a subset of predicted epithelial barrier transcripts that were previously linked to barrier integrity. miR-363-3p mimic and antagomir regulated the levels of barrier-related genes, mainly *CLDN8*, *PCDH1*, and *PTEN,* in the investigated models. We further demonstrated that miR-363-3p is increased in the airways in individuals with asthma and that miR-363-3p correlated with both airway eosinophils and neutrophils. These findings provide novel insights into a regulatory role for miR-363-3p in Th17-driven airway epithelial barrier dysfunction that may be relevant for respiratory disease pathology.

Airway epithelial integrity is essential for protecting the underlying tissue against infection, irritants, and inflammation [48], however, there are currently no direct treatments that aim to improve or restore epithelial barrier integrity in airway disease. In the current study, we show that cytokines reflective of Th17 inflammation were the most disruptive to the airway epithelium of the inflammatory milieus evaluated. The other inflammatory profiles modelled in our study caused only minor decreases in epithelial integrity, although previous studies have shown that IL-4, IL-13, TNFα, or TGFβ may decrease epithelial barrier function in the upper and lower airways [49, 50]. IL-17A has previously been suggested to have a dual role in epithelial barrier integrity [51]. While it has been shown to have a protective effect on epithelial barrier integrity in the gut [52], it has also been shown to have a disruptive effect on the nasal epithelial barrier [53]. Importantly, our findings support published data which showed that Th17 cytokines, such as IL-17, IL-22, and IL-26, but not T1 and T2 cytokines, disrupted the barrier of human nasal epithelial cells [54]. Together with our study, this emphasizes the advantages of comparing multiple conditions simultaneously in the same model system. These results align with previous observations related to Th17 inflammation and asthma, where IL-17 was associated with more severe asthma [55–57], and disease severity was often related to impaired barrier function when epithelial cells from patients were studied in vitro [58, 59].

The functional roles of miRNAs in the human airway epithelium have been examined previously [16, 19, 48]. However, only a handful of studies have assessed the involvement of miRNAs in the regulation of epithelial barrier function, with few exploring the role of their direct mRNA targets [60–63]. In bronchial epithelial cells from asthma patients, increased levels of miR-221 targeted and downregulated SIRT1, leading to apoptosis and a dysfunctional barrier [60]. In another study, miR-221 correlated with lung function in asthma patients and was suggested to be used as a biomarker of oral corticosteroid treatment [64]. miR-146a has been described to play crucial roles in maintenance of the tight junction barrier and innate immune defense protecting against invading pathogens acting as a negative regulator of TLR3-mediated inflammatory responses via direct targeting TRAF-6 in human nasal epithelial cells [63].

In this study, we identified two candidates, miR-146a-3p and miR-363-3p, that were consistently increased across multiple donors under Th17-mimicking conditions. There is currently little known about the effects of Th17 inflammation on these two miRNAs, but one study showed that levels of miR-146a correlate with blood Th17 cells in rheumatoid arthritis patients [65]. However, it was not specified whether it was miR-146a-3p or −5p that was analyzed. Furthermore, miR-146a has been associated with asthma and allergic diseases [66] and is increased in response to inflammatory stimuli such as TNF-α [67]. Interestingly, in our study miR-146a-3p and miR-363-3p were only increased in response to stimulation with IL-17A + TNFα and not with any of the other stimuli used, highlighting a possible unique miRNA signature associated with Th17 inflammation in the airway epithelium.

Importantly, based on database analyses, miR-146a-3p and miR-363-3p were predicted to target a total of 13 barrier-related genes, of which eight, *CLDN8*, *DSP*, *JAM3*, *CDH1, MPDZ*, *PCDH1*, *PTEN,* and *TJP1*, were shown to be downregulated in response to treatment with the Th17 cytokines. The Th17 cytokines affected the expression of miRNAs and their target barrier-related mRNAs, as well as the barrier permeability, suggesting that these miRNAs and their predicted targets might be involved in producing the observed phenotype. To further investigate the contribution that the two miRNAs may exert on IL-17A + TNFα-induced barrier damage, pull-down and mechanistic experiments using miRNA mimics and antagomirs were performed. The pull-down indicated that miR-363-3p targeted the analyzed transcripts *DSP* and *PTEN*, and to a lesser extent *CLDN8*, *MPDZ*, *PCDH1*, and *TJP1*. Transfection experiments with a miR-363-3p mimic decreased the expression of most of the transcripts pulled down by this miRNA, namely *CLDN8*, *PCDH1*, and *PTEN*. On the other hand, treatment of IL-17A + TNFα-stimulated epithelial cells with a miR-363-3p antagomir partially restored the cytokine-induced decrease of several of these barrier-related genes. Interestingly, genes that were decreased upon the treatment with miR-363-3p mimic in vitro*,* such as *CLDN8* and *PCDH1,* have been suggested to be involved in the regulation of epithelial function in asthma [68, 69]. Furthermore, our data suggest that miR-363-3p is increased in the airways of individuals with asthma and that it is associated with infiltrating immune cells in the airways of asthmatics, such as eosinophils and neutrophils. Limited information is available on miR-363-3p in relation to immune cells; however, one study demonstrated that both miR-363-3p levels and infiltrating neutrophils in the lungs are increased in a mouse model of acute lung injury, and that neutrophil infiltration was reduced upon administration of a miR-363-3p antagomir [70].

Although our study includes small donor numbers and a lack of in vivo validation, this is the first study to identify miR-363-3p as a potential contributor to epithelial barrier integrity in Th17-driven respiratory disease. Previous studies have shown that miR-363-3p can inhibit cell proliferation in epithelial carcinomas [71, 72], and miR-363-3p has also been linked to epithelial-to-mesenchymal transition, however the results were contradictory [73, 74]. Interestingly, miR-363-3p has been suggested to regulate Th17 cell differentiation by targeting key transcription factors of Th17 cell differentiation [75]. These findings, combined with our results demonstrating that miR-363-3p regulates barrier-related genes, suggests that miR-363-3p may have a novel, dual role in Th17-driven asthma pathogenesis, affecting both the epithelium and the immune cell compartments. Indeed, our study supports that miR-363-3p may have a regulatory role in Th17-dependent airway inflammation, which may involve both neutrophil and eosinophil-driven inflammatory pathways.

## Conclusions

To conclude, our study supports the hypothesis that miRNAs are involved in airway epithelial barrier dysfunction. We have identified a novel role for miR-363-3p as a miRNA that targets and decreases the level of several genes involved in the airway epithelial barrier. Further studies are necessary to investigate whether the relationship between miRNA and barrier transcripts holds true, and whether this can be translated to epithelial barrier function in patients with Th17-driven airway disease.

## Supplementary Information

Supplementary Material 1: Supplementary Figure 1. Cytokine stimulation alters mRNA expression in airway epithelial cells. NGS was used to identify mRNAs in HBEC-ALI influenced by the cytokines representative of different inflammatory endotypes. A) Principal component analysis (PCA) from R Studio using the "prcomp" function based on log2-transformed normalized counts of the technical replicates from the NGS of mRNA. B-E) Database for Annotation, Visualization and Integrated Discovery (DAVID) was used to determine the enriched biological processes associated with the genes induced after IL-17A+TNFα (B), IL-4+IL-13 (C), IL-6+sIL6R (D), and TGFβ (E). The 20 most enriched GO terms (based on p-value) for each stimulation are displayed. Green; IL-17+TNFα, Pink; IL-4+IL-13, Yellow; IL-6+sIL-6R, Blue; TGFβ. Supplementary Figure 2. Cytokine stimulation alters miRNA expression in airway epithelial cells. NGS was used to identify miRNAs in HBEC-ALI influenced by cytokines representative of different inflammatory endotypes. A) Principal Component Analysis (PCA) from R Studio using the "prcomp" function based on log2-transformed normalized counts of the technical replicates from the NGS of miRNA. B) The number of uniquely and mutually downregulated miRNAs in the four cytokine stimulations. Green; IL-17+TNFα, Pink; IL-4+IL-13, Yellow; IL-6+sIL-6R, Blue; TGFβ.

Supplementary Material 2: Supplementary Table 1. Cytokines used for the stimulations included in the study. Supplementary Table 2. Details for the regents used for the Taqman assay and qPCR analysis of RNA from ALI cultured bronchial epithelial cells. Supplementary Table 3. Details for the regents used in the pulldown, miRNA mimics, and miRNA antagomir experiments. Supplementary Table 4. Details for the regents used for the qPCR analysis of RNA from bronchial lavage. Supplementary Table 5. Epithelial barrier-related genes and their expression under IL-17A+TNFα stimulation versus non-stimulated control.

## Data Availability

The datasets used and/or analyzed during the current study are available from the corresponding author on reasonable request.

## Abbreviations

ALI: Air-liquid interface
BL: Bronchial lavage
CLDN: Claudin
COPD: Chronic obstructive pulmonary disease
CXCL: Chemokine (C-X-C motif) ligand
DSP: Desmoplakin
FITC: Fluorescein isothiocyanate
HBECs: Human bronchial epithelial cells
IL: Interleukin
JAM3: Junctional adhesion molecule C
miRNAs: MicroRNAs
MPDZ: Multiple PDZ domain protein
NGS: Next-generation sequencing
PCDH1: Protocadherin-1
PTEN: Phosphatase and tensin homolog
sIL-6R: Soluble IL-6 receptor
T2: Type 2
TGFβ: Transforming growth factor beta
Th17: Type 17 helper T cell
TJP1: Tight junction protein ZO-1
TNFα: Tumor necrosis factor alpha
