# Supplementary figures and images for: The involvement of Th17 inflammation and miR-363-3p in airway epithelial barrier dysfunction
Source: Respir Res. 2026 Jan 16;27:30. doi: 10.1186/s12931-025-03492-3 (PMC12849667; doi:10.1186/s12931-025-03492-3)

Supplementary Figure 1

A

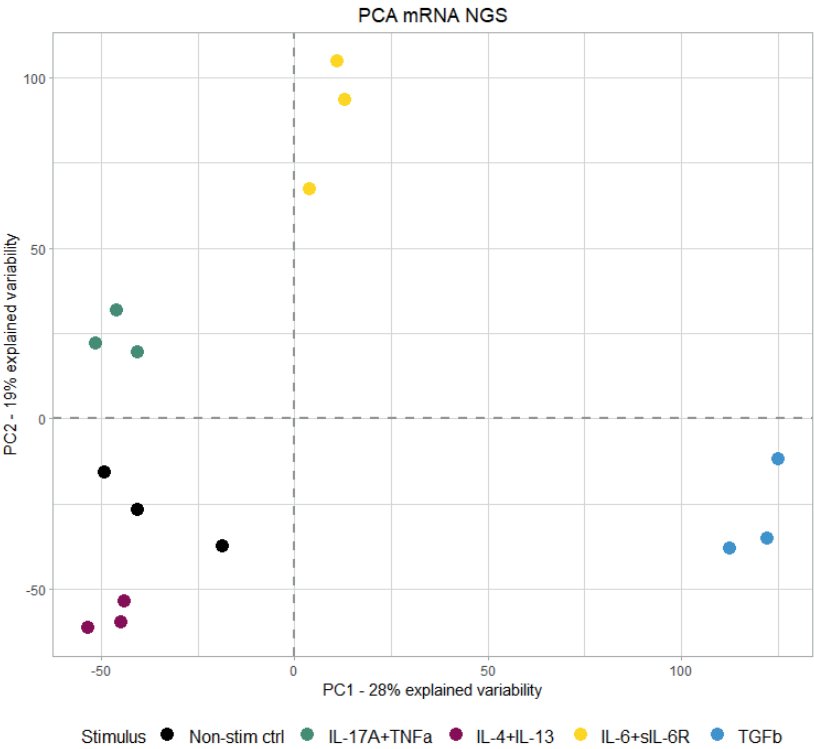

B

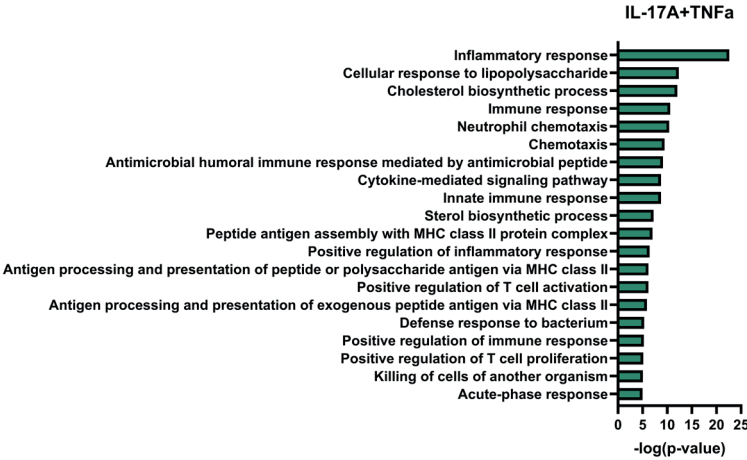

C

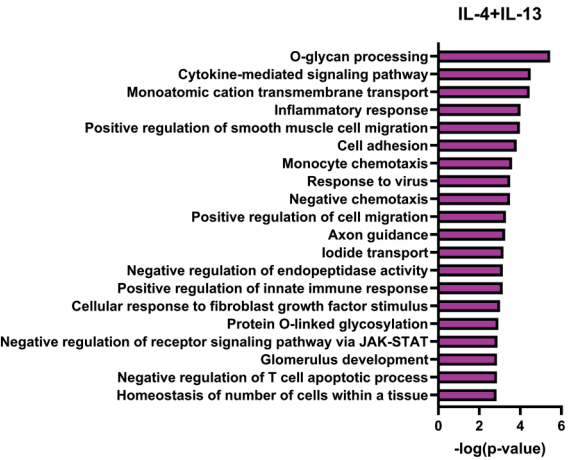

D

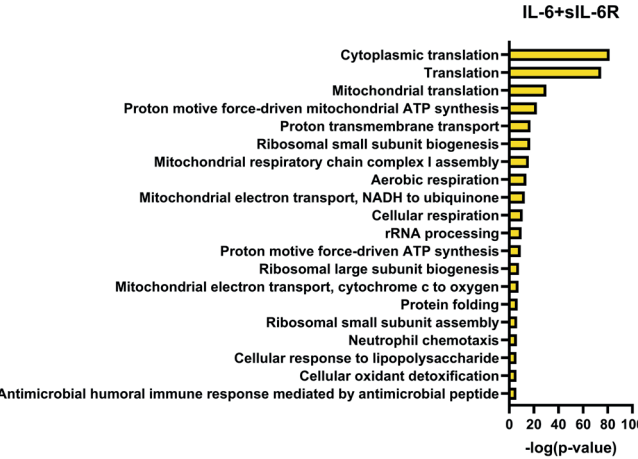

E

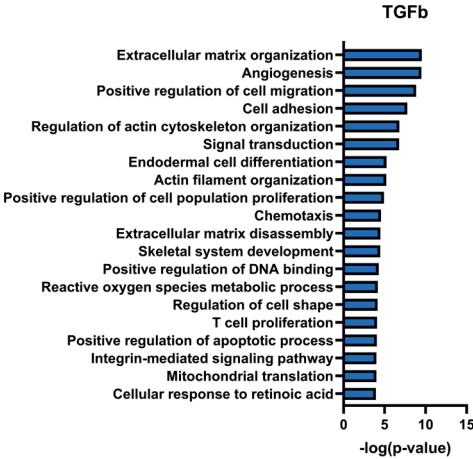

Supplementary Figure 2

A

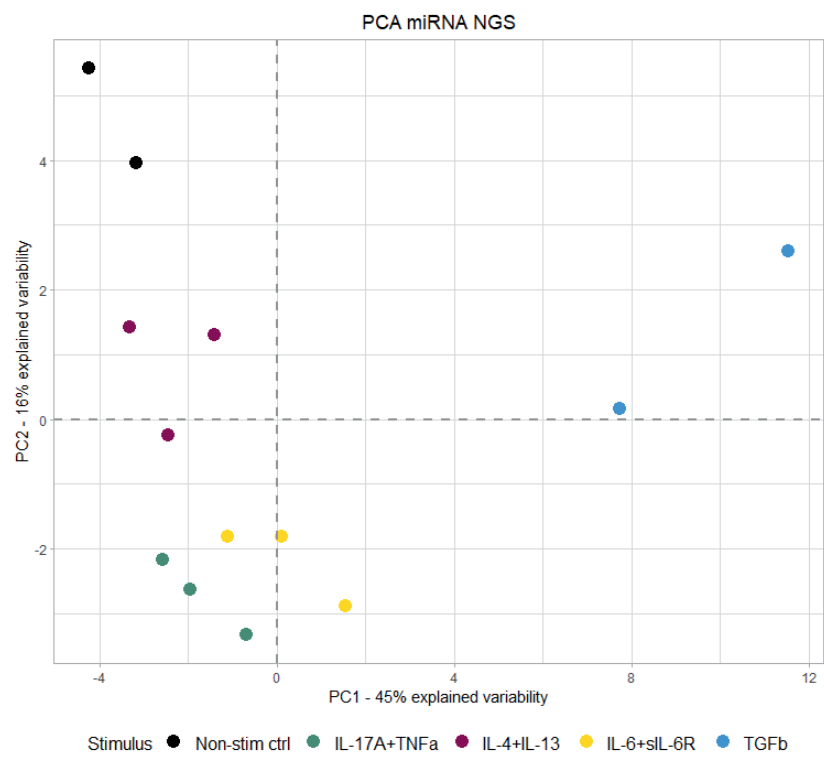

B

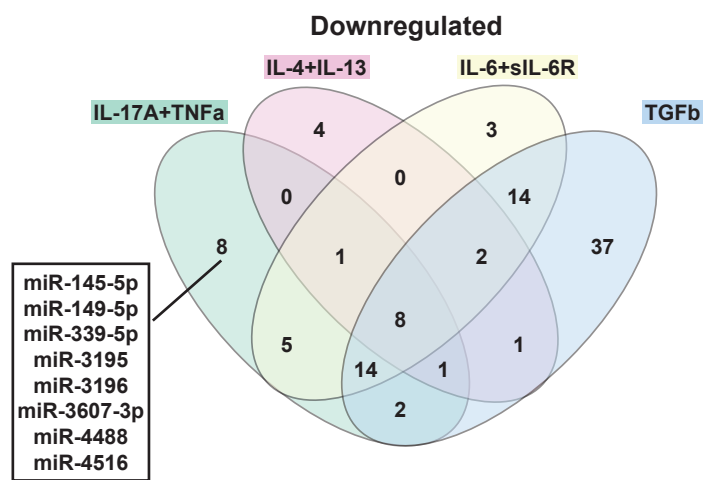

Supplement: Supplementary file 1 — Supplementary Material 1: Supplementary Figure 1. Cytokine stimulation alters mRNA expression in airway epithelial cells. NGS was used to identify mRNAs in HBEC-ALI influenced by the cytokines representative of different inflammatory endotypes. A) Principal component analysis (PCA) from R Studio using the "prcomp" function based on log2-transformed normalized counts of the technical replicates from the NGS of mRNA. B-E) Database for Annotation, Visualization and Integrated Discovery (DAVID) was used to determine the enriched biological processes associated with the genes induced after IL-17A+TNFα (B), IL-4+IL-13 (C), IL-6+sIL6R (D), and TGFβ (E). The 20 most enriched GO terms (based on p-value) for each stimulation are displayed. Green; IL-17+TNFα, Pink; IL-4+IL-13, Yellow; IL-6+sIL-6R, Blue; TGFβ. Supplementary Figure 2. Cytokine stimulation alters miRNA expression in airway epithelial cells. NGS was used to identify miRNAs in HBEC-ALI influenced by cytokines representative of different inflammatory endotypes. A) Principal Component Analysis (PCA) from R Studio using the "prcomp" function based on log2-transformed normalized counts of the technical replicates from the NGS of miRNA. B) The number of uniquely and mutually downregulated miRNAs in the four cytokine stimulations. Green; IL-17+TNFα, Pink; IL-4+IL-13, Yellow; IL-6+sIL-6R, Blue; TGFβ. [file 12931_2025_3492_MOESM1_ESM.pdf]
