# Supplementary material for: The involvement of Th17 inflammation and miR-363-3p in airway epithelial barrier dysfunction
Source: Respir Res. 2026 Jan 16;27:30. doi: 10.1186/s12931-025-03492-3 (PMC12849667; doi:10.1186/s12931-025-03492-3)
Supplement: Supplementary file 2 — Supplementary Material 2: Supplementary Table 1. Cytokines used for the stimulations included in the study. Supplementary Table 2. Details for the regents used for the Taqman assay and qPCR analysis of RNA from ALI cultured bronchial epithelial cells. Supplementary Table 3. Details for the regents used in the pulldown, miRNA mimics, and miRNA antagomir experiments. Supplementary Table 4. Details for the regents used for the qPCR analysis of RNA from bronchial lavage. Supplementary Table 5. Epithelial barrier-related genes and their expression under IL-17A+TNFα stimulation versus non-stimulated control. [file 12931_2025_3492_MOESM2_ESM.docx]

**Supplementary Table 1. Cytokines used for the different stimulations.**

| Cytokine | Concentration | Source | Catalog number |
| --- | --- | --- | --- |
| IL-4 | 30 ng/ml | R&D Systems | 204-IL |
| IL-13 | 30 ng/ml | R&D Systems | 213-ILB |
| IL-17A | 30 ng/ml | R&D Systems | 317-ILB |
| TNFα | 30 ng/ml | R&D Systems | 210-TA |
| IL-6 | 150 ng/ml | PeproTech | 200-06 |
| sIL-6R | 150 ng/ml | PeproTech | 200-06R |
| TGFb1 | 150 ng/ml | R&D Systems | 240-B |

**Supplementary Table 2. Details for the regents used for the Taqman assay and qPCR on RNA from ALI cultured bronchial epithelial cells**

| Reagent | Source | Catalog number | Target | Assay ID |
| --- | --- | --- | --- | --- |
| Taqman assay, miRNAs | Thermo Fisher Scientific | A25576 | let-7a-3p | 477861_mir |
|  |  |  | miR-9-5p | 478214_mir |
|  |  |  | miR-19a-3p | 479228_mir |
|  |  |  | miR-21-3p | 477973_mir |
|  |  |  | miR-28-3p | 477999_mir |
|  |  |  | miR-32-3p | 478827_mir |
|  |  |  | miR-34c-3p | 478051_mir |
|  |  |  | miR-101-3p | 477863_mir |
|  |  |  | miR-146a-3p | 478714_mir |
|  |  |  | miR-186-5p | 477940_mir |
|  |  |  | miR-363-3p | 478060_mir |
|  |  |  | miR-429 | 477849_mir |
|  |  |  | miR-590-5p | 478367_mir |
| Taqman assay, mRNAs | Thermo Fisher Scientific | 4331182 | ACTB | Hs99999903_m1 |
|  |  |  | CDH1 | Hs01023895_m1 |
|  |  |  | CGN | Hs00430426_m1 |
|  |  |  | CLDN1 | Hs00221623_m1 |
|  |  |  | CLDN8 | Hs04186769_s1 |
|  |  |  | DSP | Hs00950591_m1 |
|  |  |  | JAM3 | Hs00230289_m1 |
|  |  |  | MPDZ | Hs00187106_m1 |
|  |  |  | PCDH1 | Hs00170174_m1 |
|  |  |  | PTEN | Hs02621230_s1 |
|  |  |  | TJP1 | Hs01551861_m1 |

**Supplementary Table 3.** **Details for the regents used in the pulldown, miRNA mimics, and miRNA antagomir experiments.**

| Reagent | Source | Catalog number | Target | Assay ID |
| --- | --- | --- | --- | --- |
| Biotin-tagged miRNA mimics | QIAGEN | 339178 | miR-146a-3p | YM00472210-BDI |
|  |  | 339178 | miR-363-3p | YM00470786-BDI |
|  |  | 339178 | Negative ctrl | YM00479902-BDI |
| miRNA mimics | QIAGEN | 339173 | miR-363-3p | YM00470786-ADA |
|  |  | 339173 | Negative ctrl | YM00479902-ADA |
| miRNA antagomirs | QIAGEN | 339131 | miR-363-3p | YI04101050-DDA |
|  |  | 339136 | Negative ctrl | YI00199006-DDA |

**Supplementary Table 4. Details for the regents used for the RT-qPCR on RNA from bronchial lavage**

| **Reagent** | **Source** | **Catalog number** | **Target** | **Assay ID** |
| --- | --- | --- | --- | --- |
| miRCURY LNA SYBR Green PCR | Qiagen | 339345 | miR-363-3p | YP00204726 |
|  |  |  | miR-103a-3p | YP00204063 |
|  |  |  |  |  |

**Supplementary Table 5. Epithelial barrier-related genes and their expression under IL-17A+TNFα stimulation versus non-stimulated control (n=1).** FC: fold change, -: not significantly altered.

| Protein name | Gene name | Junction | log2(FC) |
| --- | --- | --- | --- |
| E-cadherin | CDH1 | Adherens | -0.58 |
| N-cadherin | CDH2 | Adherens | - |
| P-cadherin | CDH3 | Adherens | - |
| Cingulin | CGN | Tight | -0.49 |
| Claudin 1 | CLDN1 | Tight | -0.67 |
| Claudin 2 | CLDN2 | Tight | - |
| Claudin 3 | CLDN3 | Tight | - |
| Claudin 4 | CLDN4 | Tight | - |
| Claudin 5 | CLDN5 | Tight | 1.58 |
| Claudin 7 | CLDN7 | Tight | 0.49 |
| Claudin 8 | CLDN8 | Tight | -2.49 |
| Claudin 9 | CLDN9 | Tight | -1.13 |
| Claudin 10 | CLDN10 | Tight | 1.17 |
| Claudin 16 | CLDN16 | Tight | - |
| Catenin alpha-like-1 | CTNNAL1 | Adherens | - |
| Catenin beta-1 | CTNNB1 | Adherens | - |
| Catenin, beta interacting protein 1 | CTNNBIP1 | Adherens | - |
| p120 catenin/Catenin delta-1 | CTNND1 | Adherens | - |
| Coxsackie virus and adenovirus receptor | CXADR | Tight | - |
| Desmocollin-1 | DSC1 | Desmosome | - |
| Desmocollin-2 | DSC2 | Desmosome | - |
| Desmocollin-3 | DSC3 | Desmosome | -1.17 |
| Desmoplakin | DSP | Desmosome | -0.65 |
| Jam-A/1 | F11R | Tight | - |
| Gap junction beta-2 protein/Connexin 26 | GJB2 | Gap | 1.56 |
| Jam-B/2 | JAM2 | Tight | - |
| Jam-C/3 | JAM3 | Tight | -1.92 |
| Plakoglobin/Gamma-catenin | JUP | Desmosome | - |
| Membrane associated guanylate kinase, WW and PDZ domain containing 1 | MAGI1 | Tight | -0.54 |
| Membrane associated guanylate kinase, WW and PDZ domain containing 2 | MAGI2 | Tight | - |
| Membrane associated guanylate kinase, WW and PDZ domain containing 3 | MAGI3 | Tight | - |
| Tricellulin | MARVELD2 | Tight | - |
| Afadin/AF6 | MLLT4 | Adherens | - |
| Multiple PDZ domain protein | MPDZ | Tight | -0.53 |
| Membrane protein, palmitoylated 1 | MPP1 | Tight | - |
| Membrane protein, palmitoylated 7 | MPP7 | Tight | - |
| Nectin-1 | NECTIN1/PVRL1 | Adherens | -0.94 |
| Nectin-2 | NECTIN2/PVRL2 | Adherens | - |
| Nectin-3 | NECTIN3/PVRL3 | Adherens | - |
| Occludin | OCLN | Tight | - |
| Protocadherin-1 | PCDH1 | Adherens | -0.58 |
| Phosphatase and tensin homolog | PTEN | Other | -0.32 |
| Protein tyrosine phosphatase, receptor type, M | PTPRM | Adherens | - |
| Symplekin | SYMPK | Tight | - |
| ZO-1 | TJP1 | Tight | -0.35 |
| ZO-2 | TJP2 | Tight | - |
| ZO-3 | TJP3 | Tight | 0.61 |
| Vinculin | VCL | Adherens | - |
| WNK lysine deficient protein kinase 4 | WNK4 | Tight | - |
| Cold shock domain protein A | YBX3 | Tight | - |
